# Prevalence of migraine headaches and their impact on the academic performance of Sudanese medical students using ID‐Migraine test as a screening tool: A cross‐sectional study

**DOI:** 10.1002/brb3.2588

**Published:** 2022-04-21

**Authors:** Moaid Mohamed Osman Ali, Khabab Abbasher Hussien Mohamed Ahmed, Mohammed Eltahier Abdalla Omer

**Affiliations:** ^1^ Faculty of Medicine University of Khartoum Khartoum Sudan; ^2^ Faculty of Medicine and Health Sciences, Gadarif University Al Qadarif Sudan

**Keywords:** academic performance, headache, migraine, students, Sudan

## Abstract

**Background:**

Migraine—and episodic headache—is one of the most common types of primary headache. Migraine is considered a serious health problem that affects the quality of life. During university life, students often report increased levels of stress, depression, anxiety, and irregular sleep, all of which are associated with migraines. Our aim was to determine the prevalence of migraine headaches among medical campus students at the University of Khartoum, Sudan. Based on available data, migraine is on the rise in both general populations as well as in university students.

**Methods:**

This is a cross‐sectional study. The study population was composed of students registered to the Faculty of Medicine, Dentistry, and Pharmacy at Khartoum University in the academic years of 2020–2021. Out of these, 318 of them accepted to participate. Participants who had two or more headaches in the last 3 months formed the headache group. Afterwards, two preliminary questions were applied to the headache group and participants with at least one affirmative response were asked to perform the validated ID‐Migraine™ test.

**Results:**

The mean age of 318 students participating in the study was 19.23 ± 1.84 (17–39 years), with adolescents:adult ratio being 2.5:1. A total of 1613 students (43.7%) had at least two headaches in the last 3 months. Migraine‐type headache was detected in 266 subjects (7.2%) based on the ID‐Migraine™ test. Of the migraine group, 72 were male (27.1%) and 194 were female (72.9%). There was no significant difference in migraine prevalence between adolescent and adult age groups.

**Conclusions:**

With prevalence similar to adults, primary care physicians should be aware of the probability of migraine headaches in university students in order to maintain a successful school performance.

## Abstract 2

1. Migraine—and episodic headache—is one of the most common types of primary headache.

2. Migraine is considered a serious health problem that affects the quality of life.

3. During university life, students often report increased levels of stress, depression, anxiety, and irregular sleep, all of which are associated with migraines. Our aim was to determine the prevalence and effect of migraine headaches on academic performance among medical campus students at University of Khartoum, Sudan.

## 1 BACKGROUND

Primary headache disorders are a diverse group of neurologic conditions that cause recurrent or persistent headaches with no obvious cause (Robbins & Lipton, 2010). Migraine is being recognized as a significant health problem affecting the quality of life and a major cause of morbidity among young adults specially students, and one of the major health‐related resource consumers. It is a chronic neurological disease that affects 15% of the population, but there is little information about its prevalence in Sudan (Halay et al., 2021). During university life, students often report increased levels of stress, depression, anxiety, and irregular sleep, all of which are associated with migraines. Migraine is on the rise in both general populations and university students, according to available statistics. The activation of the so‐called trigeminovascular system (TGVS) causes it: trigeminal afferents activate structures involved in pain transmission and perception, as well as the release of vasoactive peptides (presumably causing neurogenic inflammation). The primary cause of TGVS activation and the mechanism of pain generation after TGVS activation are the two major open questions in migraine headache neurobiology. Regarding the main cause of TGVS activation, two theories have dominated the field: cortical spreading depression (a slowly moving wave of cortical depolarization) and the existence of a brainstem generator (dysfunctional brainstem nuclei involved in antinociception as a primary cause of migraine). In terms of migraine pain mechanisms, two main theories have been proposed: neurogenic inflammation of the meninges and trigeminal nerve and nucleus sensitization. There is a strong genetic component to migraine. CACNA1A (which encodes a subunit of the voltage‐gated Ca2+ channel CaV2.1) and ATP1A2 (which encodes a subunit of the Na+/K+ ATPase) are the only two genes known to cause a rare form of migraine. The discovery of migraine‐linked mutations, as well as the identification of the most likely primary cause of TGVS activation, has provided us with new targets for the development of antimigraine compounds. Due to the prevalence of the disease and the fact that currently available drugs are not always successful, this is a critical area (May & Goadsby, 1999; Moskowitz & MacFarlane, 1993; Noseda & Burstein, 2021; Wessman et al., 2002). Migraine headaches have a wide range of prevalence rates. The prevalence of migraine‐type headaches varies depending on the technique used to diagnose them in different studies. There have been few studies on the prevalence of migraine in Sudanese university students. It is particularly significant among university students because of its negative effects on quality of life. World Health Organization (WHO) must maintain a high level of concentration and achievement. Headaches have a significant effect on university students' academic performance. Students with migraines have a 62.7% decrease in capacity compared to students with episodic tension‐type headaches (ETTH), who have a 24.4% decrease in capacity. Furthermore, students suffering from migraine headaches missed more school than those suffering from ETTH.

The significance of migraine headaches in university students is revealed by these findings. To ascertain the prevalence, large populations must be subjected to useful, reliable, and validated screening tests. Lipton et al. (2016) developed and validated the Identity Migraine (ID Migraine TM) test, which is a proper and useful screening tool that can be applied quickly to large populations. In primary care, this test's sensitivity, specificity, and positive predictive value have been determined to be 81%, 75%, and 93%, respectively. Adolescence is defined by the WHO as young people aged 10 to 19. The university population includes both teens and adults (Deleu et al., 2021). With a sensitivity of 62.1% and a specificity of 71.1%, the test has been validated for use among adolescent students (Deleu et al., 2021; El‐Metwally et al., 2021; Zarifoğlu et al., 2021). In this study, our goal is to use the ID Migraine™ rapid detection tool to determine the prevalence of migraine in college students. The prevalence of migraine in the general population ranged from 2.6% to 32%. The prevalence of migraine headache was estimated to be between 12.2% and 27.9% among medical university students and between 7.1% and 13.7% among school children (6 to 18 years). Females were found to be more likely than males to suffer from migraines. The duration of migraine attacks became shorter as people got older, while chronic (daily) migraine became more common. Anxiety, hypertension, irritable bowel syndrome, and depression were the most frequently observed comorbidities with migraine. Stress, fatigue, sleep disturbances, prolonged exposure to excessive sunshine or heat, and starvation were the most common headache‐inducing factors. The prevalence and risk factors of migraine headache in Arab countries are comparable to reports from the western countries. Longitudinal studies are still needed to examine the prognosis and predictors of chronic diseases in Arab countries (Wang et al., 2015).

Migraine is a major cause of morbidity among young adults especially students, and one of the major health‐related resource consumers. Migraine is a very important disease, and there is no enough researches done for it and is easily preventable—but at the same time it affects quality of life such as students’ academic performance—by proper practice of consuming good prescribed drugs, so this research will help primary healthcare providers in the effort to decrease adult students’ morbidity.

## 2 OBJECTIVES

### 2.1 General objectives

- To determine the prevalence of migraine among young students in the medical campus at University of Khartoum and the impact on their academic performance.

### 2.2 Specific objectives

1. To find out the impact on academic performance of those having the disease.
2. To determine the most affected gender and age.
3. To assess family association.

## 3 METHODS AND MATERIALS

### 3.1 Study design

It was a university‐based cross‐sectional study.

### Study area

The medical campus at the University of Khartoum, which contains three faculties (medicine, dentistry, and pharmacy), is located in El Qasr Ave, Khartoum, Sudan.

### 3.2 Study population

All medical campus students at the University of Khartoum.

### 3.3 Inclusion criteria

 

1. All medical campus students in the three faculties (medicine, dentistry, and pharmacy), males and females in all batches at the University of Khartoum.
2. Willing to take part in the study.

### 3.4 Exclusion criteria

 

1. Postgraduate students at the medical campus, University of Khartoum.

### 3.5 Sampling techniques

Probability sampling: systematic random sampling. All cases (all batches of medical campus students who are diagnosed by migraine using ID‐Migraine test as a screening tool) are available at medical campus in University of Khartoum.

### 3.6 Sample frame

Simple random sampling technique was used.

### 3.7 Sample size

All cases are available in the medical campus of the University of Khartoum in the period of data collection.

Equation used:

$$no=\frac{Z^{2}pq}{e^{2}}$$

where: *no* = initial sample size required, *z* = probability that *e* is not exceeded (*e* score of 1.645 corresponds to 90% confidence level), *p* = expected prevalence (estimated as .5), *q* = 1−*p*, *e* = maximum acceptable random sampling error (here is 5%).

So my initial sample size (*no*) is = .67650625/(5%) = 270.

So by this equation: the last sample size *n* = *no*/(1+(*no*/*N*)), where *N* is the population size, which is 3814 medical campus students, it was found to be 253 samples from the study population.

### 3.8 Data collection methods

Structured interviews using pretested close‐ended self‐administered questionnaire.

### 3.9 Data collection tools

Interviews by self‐prepared questionnaires that include questions about sociodemographic status, age, and gender, and so forth were taken from the candidates (ID‐Migraine test as a screening tool was used). A questionnaire was constructed based on our objectives, and the literature was previewed. Due to Covid‐19 restrictions, all questionnaires were distributed online using Google form and through social media platforms. Mostly, WhatsApp and Telegram were used.

The questionnaire consisted of eight sections;

- Demographic information.
- Migraine according to MS‐Q.
- Questions for determination of probability migraine‐type headache: preliminary questions.
- Determination of migraine‐type headache: three items of ID‐Migraine test.
- Headache severity assessment.
- Triggering factors.
- Family correlation.
- Academic performance evaluation.

## 4 STUDY VARIABLES

Independent variables: gender, age, weight, height, and study faculty.

Dependent variables: Migraine according to MS‐Q questions for determination of probability of migraine‐type headache (preliminary questions), three items of ID‐Migraine test, academic performance, headache severity assessment questions triggering factors, and family correlations.

### 4.1 Data entry and analysis

Microsoft Excel was used for data entry and SPSS v.25 for data analysis.

Frequencies and pie/bar charts were used for descriptive data. Pearson chi‐square test was used for inferential data.

### 4.2 Ethical consideration

Ethical approval was taken from the Department of Community Medicine, University of Khartoum. Both verbal and written consents were taken from each individual. Each individual was voluntarily participating. Each individual had the right of withdrawal at any time of the study. High degree of confidentiality was preserved.

## 5 RESULTS

At the end of the study, out of 3814 students, 270 were supposed to be collected, but fortunately, the data were collected from 318 participants and were analyzed. Of the participants, 15.72% were male (*n* = 50) and 83.81% female (*n* = 259). The mean age of the participants was 21.64 ± 1.84, ranging from 17 to 28 years of age. Two hundred and fifty‐two students (79.2%) who replied “yes” to the question “Did you have two or more headaches in the last 3 months?” formed the headache group. Of the headache group, 12.69% were male (*n* = 32) and 87.3% female (*n* = 220). Female participants had significantly higher headache rates than male ones (Pearson χ^2^ = 35.344, *p* < .001).

As preliminary questions were analyzed, 157 participants of the headache group (49.4%) had at least one positive response. Eighty‐one participants (25.5%) had “a desire to talk to a healthcare professional about these headaches” and 150 participants expressed that “his/her ability to work, study, or enjoy life was limited” (47.2%).

This group of participants was further evaluated in terms of migraine‐type headaches with application of the three‐item ID Migraine™ test. Of this group, 34.3% (*n* = 109) gave at least two positive responses and had positive ID Migraine™ tests. The prevalence of migraine among all participants according to the ID Migraine™ test was 2.9%. Of the migraine group, 14 were male (12.8%) and 95 were female (87.2%). The rate of migraine in female participants was found to be significantly higher than male ones (Pearson χ^2^ = 60.725, *p* < .001. The mean age of the participants with migraine was 21.72 ± 1.91, ranging from 17 to 28 years of age. There was no statistical significance between the ages of participants and migraine prevalence (Pearson χ^2^ = 16.958, *p* = .593). Of the participants, 14.7% (*n* = 16) were in the adolescent age, whereas 85.3% (*n* = 93) were in the adult group, with a ratio of 1:6. In comparison, there was a significant difference between adolescent and adult age groups concerning the migraine rates (7.0% and 7.8%, respectively). In addition, it shows the prevalence according to gender.

In the migraine group (*n* = 109), three‐item ID Migraine™ test showed 56% of the participants (*n* = 61) “felt nauseated or sick to the stomach,” 82.6% of the participants (*n* = 90) were “bothered by light (a lot more than when they don't have headaches),” and 94.5% (*n* = 103) expressed that “their headaches limited their ability to work, study or do what they needed to do for at least one day.” The headache severity assessment was done and found to be that 11.9% of the participants (*n* = 13) were experiencing “mild headache,” 30.3% of the participants (*n* = 33) expressed that they were suffering from “severe headache,” and 57.8% of the participants (*n* = 63) were having “moderate headache.” Headache‐triggering factors were also been asked and 21.1% of the participants (*n* = 23) were triggered by “emotional stress or anxiety,” 23.9% of the participants (*n* = 26) were triggered by “eating habits,” 24.8% of the participants (*n* = 27) were triggered by “fasting,” 64.2% of the participants (*n* = 70) were triggered by irregular sleep, 33.9% of the participants (*n* = 37) were triggered by “physical activity,” 39.5% of participants (*n* = 43) were triggered by “menstruation,” 58.7% of the participants (*n* = 64) were triggered by “noise,” 36.7% of the participants (*n* = 40) were triggered by “tests or exams,” 48.6% of the participants (*n* = 53) were triggered by “reading hours (when you are studying for example),” 2.8% of the participants (*n* = 3) were triggered by “smoking,” and 71.6% of the participants (*n* = 78) were triggered by “exposure to sun.” The question of the family correlation had been asked, and 28.4% of the participants (*n* = 31) were having at least part of their families diagnosed by migraine.

### 5.1 Impact of headaches on academic life

The majority of participants (74.3%) continued attending lectures while experiencing a headache. A large portion of the participants stated that headaches affected studying for tests and/or examinations (92.7%). Majority reported that experiencing a headache limited their concentration at lectures (97.2%; *p* < .001) and felt too tired to continue working (98.2%; *p* < .001). Almost more than half of the participants (53.2%) indicated that the headache was more intense than usual when studying for tests and exams (*p* < .001). Almost more than a third of participants that experienced a headache when studying, stopped studying due to the headache (33.9%), and some (8.3%) continued without the use of medication. However, a significant proportion (57.8%) continued with the use of medication (*p* < .001).

Sleeping patterns were altered during tests and/or examination periods (90.8%; *p* < .001). More than a third of the participants studied for long periods without taking regular breaks (42.2%). A large number of participants consumed beverages such as caffeinated energy drinks, chocolate, or coffee (66.1%) to help sustain their concentration for a longer period of time. Consumption of these drinks during a headache made the study session less effective (*p* < .001). Lighting in the study area was adequate and did not affect studying (75.2 %; *p* < .001).

## 6 DISCUSSION

This study has been conducted among adolescents and adults registered to different faculties of medical campus at Khartoum University, Khartoum, Sudan. The ID Migraine™ test has been used for screening migraine‐type headaches. ID Migraine™ check showed that 2.9% (*n* = 109) of the 3814 students had hemicrania sort headache. The prevalence of migraine is reported to be the highest in females rather than males. However, it is also six times more among adults and university students, a significant difference between adolescent and adult populations. There are few studies focusing on migraine among university students in Sudan showing differences in prevalence. Halay et al. (2021) reported a prevalence of 40% of those who had at least two headache episodes. Females were more affected by migraine than males, and the most common triggering factors were irregular/lack of sleep, stress/anxiety, noise, and fatigue/physical activity, which accounted for 91.0%, 88.0%, 85.7%, and 84.6% of the population with migraine, respectively. Menstruation‐related migraines were reported by 46.7% of females. The impact of migraine on work, everyday activities, and leisure was mild to extreme for 78.2% of migraine sufferers, but in this study, 21.1% of the participants were triggered by “emotional stress or anxiety,” 23.9% of the participants were triggered by “eating habits,” 24.8% of the participants were triggered by “fasting,” 64.2% of the participants were triggered by irregular sleep, 33.9% of the participants were triggered by “physical activity,” 39.5% of participants were triggered by “menstruation,” 58.7% of the participants were triggered by “noise,” 36.7% of the participants were triggered by “tests or exams,” 48.6% of the participants were triggered by “reading hours (when you are studying for example),” 2.8% of the participants were triggered by “smoking,” and 71.6% of the participants were triggered by “exposure to sun.” In some international studies, for example, in Nigeria, 1679 students aged 11–18 years were recruited. Several studies throughout the world show different results. The prevalence of migraine among university students is reported to be 27.9% in a Kuwaiti study, 9.0% in a Chinese study among 5129 students; 7.2% in a Turkish study among 3694 students (Al‐Hashel et al., 2014; Genizi et al., 2013; Oztora et al., 2011; Wang et al., 2015). The overall prevalence of headache was 19.5% compared to my study which of 3814 about 2.9%, but the same in that prevalence rate is more in females compared to males (Ofovwe & Ofili, 2010). We found the prevalence of migraine headaches relatively lower than Sudanese and also international studies. Our relatively large number of subjects will affect the results. Since the screening test is based on the presence or absence of headaches in the past 3 months, the duration of the study may also be a limiting factor, which varies from study to study. A study conducted in more stressful periods like midterms or final examinations would have revealed a higher prevalence of migraine headaches. In addition, the method used to determine the prevalence of migraine can significantly affect the prevalence, which explains the differences observed in epidemiological studies. The female/male ratio was (7:1) (95/14) in our study, whereas in another study in Israel, it was 1.25:1 in Haifa which means that our study ratio was very high. A new finding is that most of the students in this group continued to attend classes despite their headaches. This is a typical feature of other studies that reported the same situation, but their attention was reduced, which had a negative impact on their research. Changes in sleep patterns during headache attacks make tests and examinations worse. Sleep disturbances have previously been reported to influence the frequency and duration of migraines (Basdav et al., 2016).

Although attending meetings frequently, the learning model was disrupted: most people were unable to study, many continued to learn from drugs, some dropped out of school due to headaches, and some continued to study without taking drugs. This can have a negative impact on test results and success rates due to wasted time, especially since those who continue the research report increased pain intensity during exercise. In addition, the frequency and intensity of headaches increased during the test and examination. It is related to the stress that students experience during their studies; students who drink caffeine and energy drinks while studying may stay awake longer (Goadsby et al., 2002). Although the level of energy drink intake during study periods was not calculated in this study, students should be warned about the negative consequences of drinking significant amounts of these beverages. Headache severity assessment showed that the majority of them were having moderate headaches, and this is common as well as in previous studies.

## 7 CONCLUSIONS

Migraine is a common adolescent ailment, despite the fact that it is considered to be an adult ailment. Even in neurology practice, it is still an underappreciated disorder. As a consequence, it is easy to overlook in both teens and adults. Primary care doctors should be aware of the risk of migraine headaches in order to ensure a good school outcome. The ID Migraine^TM^ test is an useful valid and quick screening tool, which will help primary care physicians in order to diagnose migraine in adolescents as well.

### 7.1 Recommendations

1. More research should be done on migraine headaches among university students in Sudan and among the population in general and concentrating especially on the impact of it on different aspects of life.
2. We recommend that all who are diagnosed by migraine should be considered as cases needed to be monitored and managed well according to standard guidelines.
3. When it comes to dealing with migraine as a disease, there is an obvious gap in the knowledge. We recommend that it be added as part of the medical curriculum.
4. The students should be made aware of the services provided by the university clinic, on a regular basis.

## CONFLICT OF INTEREST

The authors declare that they have no competing interests.

## AUTHOR CONTRIBUTIONS

All three authors participated in planning the study, data collection, data analysis, results writing, and writing the manuscript. All authors gave their consent for publication.

## Data Availability

The materials datasets used and/or analyzed during this study are available from the corresponding author on reasonable request.
